# Chloroform Externally Applied

**Published:** 1849-10

**Authors:** 


### CHLOROFORM EXTERNALLY APPLIED.

The following examples of the successful application of chloroform over the seat of pain, which have lately come under our observation, appear to us worthy of record.

Mrs. ——, a respectable lady had contracted lues from her child, to whom the disease had been communicated by a black nurse. Her physician for a long time mistook the character of the complaint both in her child and herself; but when its true nature was revealed he soon succeeded in curing the latter, as well as the nurse. We saw the lady a year after she had been under medical treatment, and at that time she had ulcers on the scalp, and was suffering from most acute neuralgic pain of the side of her head, which grew worse as the day declined and rendered her nights sleepless and wretched. We advised the local application of chloroform. Thirty drops were placed on a cloth which was kept in close contact with the head, and this was renewed from time to time until anesthesia was induced. The effect was prompt and most satisfactory. The night following the first application of the remedy was one of comfort, and the appearance of the patient, next morning, proved that she had enjoyed refreshing sleep. The use of the remedy was continued for a few nights; but before an ounce of chloroform had been consumed she was free from the pain, which, in three months, has not returned.

The character of the next case was entirely different, though the result was quite as satisfactory. A colored woman had a tooth extracted in September. The operation was followed by inflammation of the lower jaw, which extended to the tonsils, and made deglutition impracticable. She was bled, and afterwards a poultice, on which half a drachm of chloroform had been dropped, was applied to her throat. In half an hour, after the renewal once or twice of the anesthetic, she became easy, fell asleep, and when she awoke, a few hours afterwards, found that she could swallow. Remedies were then administered, and the patient has recovered. No doubt the bleeding contributed to the removal of the cause which obstructed deglutition; but we have as little doubt that chloroform also had a large share in the result.

